# Topical TWEAK Accelerates Healing of Experimental Burn Wounds in Mice

**DOI:** 10.3389/fphar.2018.00660

**Published:** 2018-06-21

**Authors:** Jing Liu, Lingling Peng, Yale Liu, Kunyi Wu, Sijia Wang, Xuening Wang, Qilu Liu, Yumin Xia, Weihui Zeng

**Affiliations:** ^1^Department of Dermatology, The Second Affiliated Hospital, School of Medicine, Xi’an Jiaotong University, Xi’an, China; ^2^Core Research Laboratory, The Second Affiliated Hospital, School of Medicine, Xi’an Jiaotong University, Xi’an, China

**Keywords:** TWEAK, Fn14, burn wound, animal model, differentiation, dermal fibroblast

## Abstract

The interaction of tumor necrosis factor-like weak inducer of apoptosis (TWEAK) and its receptor fibroblast growth factor inducible 14 (Fn14) participates in inflammatory responses, fibrosis, and tissue remodeling, which are central in the repair processes of wounds. Fn14 is expressed in main skin cells including dermal fibroblasts. This study was designed to explore the therapeutic effect of TWEAK on experimental burn wounds and the relevant mechanism underlying such function. Third-degree burns were introduced in two BALB/c mouse strains. Recombinant TWEAK was administrated topically, followed by the evaluation of wound areas and histologic changes. Accordingly, the downstream cytokines, inflammatory cell infiltration, and extracellular matrix synthesis were examined in lesional tissue. Moreover, the differentiation markers were analyzed in cultured human dermal fibroblasts upon TWEAK stimulation. The results showed that topical TWEAK accelerated the healing of burn wounds in wild-type mice but not in Fn14-deficient mice. TWEAK strengthened inflammatory cell infiltration, and exaggerated the production of growth factor and extracellular matrix components in wound areas of wild-type mice. Moreover, TWEAK/Fn14 activation elevated the expression of myofibroblastic differentiation markers, including alpha-smooth muscle actin and palladin, in cultured dermal fibroblasts. Therefore, topical TWEAK exhibits therapeutic effect on experimental burn wounds through favoring regional inflammation, cytokine production, and extracellular matrix synthesis. TWEAK/Fn14 activation induces the myofibroblastic differentiation of dermal fibroblasts, partially contributing to the healing of burn wounds.

## Introduction

Burn injuries, especially thermal burns, are frequently observed in the hospital setting. Burn wound repair is a dynamic process with overlapping phases, including initial inflammatory and subsequent proliferative phases. A period of tissue regeneration consists of epithelialization, angiogenesis and collagen accumulation in a remodeling process to restore the tissue (Mangoni et al., 2016). In third-degree burns, dermal fibroblasts serve an important role in wound healing through proliferation, differentiation, and collagen synthesis (Zheng and Yu, 2018). During tissue modeling, dermal fibroblasts are activated with excessive production of proinflammatory cytokines and growth factors (Rowan et al., 2015). Moreover, dermal fibroblasts are also more sensitive to relevant cytokines and become myofibroblasts at proliferative phase (Rowan et al., 2015). Therefore, the function of dermal fibroblasts is critical in the healing of burn wounds.

Tumor necrosis factor-like weak inducer of apoptosis (TWEAK) is a regulator of proinflammatory cytokines, and acts through binding to its receptor fibroblast growth factor-inducible 14 (Fn14). TWEAK is mainly produced by immune cells such as macrophages that infiltrate in inflamed tissue (Liu et al., 2017a). Fn14 is expressed in cutaneous resident cells including keratinocytes, dermal microvascular endothelial cells, and dermal fibroblasts; TWEAK induces the production of downstream cytokines in these cells (Liu et al., 2017a). Under normal conditions, Fn14 is weakly expressed in skin tissue and resident cells, maintaining cutaneous homeostasis. However, injured skin may excessively express both TWEAK and Fn14, which interact in these cells, regulating their functions and interactive responses (Cheng et al., 2015, 2016; Doerner et al., 2015; Sidler et al., 2017). Moreover, TWEAK/Fn14 signals may drive the progression of tissue fibrosis in other inflamed organs through activating fibroblasts (Chen et al., 2015; Xia et al., 2015). Obviously, TWEAK/Fn14 signals regulate resident cells and are necessary for tissue repair.

Recently, it was found that moderate TWEAK/Fn14 signals exhibit a protective role in cardiac wound repair by promoting myogenesis and angiogenesis (Blanco-Colio, 2014; Enwere et al., 2014). Also, we confirmed that TWEAK/Fn14 activation can enhance the expression of matrix metalloproteinase-9 (MMP-9) and a disintegrin and metalloproteinase 17 (ADAM17) in skin cells (Liu et al., 2017b), both of which are actually upregulated in marginal regions of skin wounds (Kawaguchi and Suzuki, 2014; Kopcewicz et al., 2017). Furthermore, TWEAK induces the differentiation of keratinocytes in skin as well as fibroblasts in other tissues (Peternel et al., 2011; Huang et al., 2014). By taking into consideration that inflammatory responses, angiogenesis, and cell differentiation are key processes during epidermal regeneration and skin remodeling, we presume that TWEAK/Fn14 signals may also function in the healing of burn wounds. The purpose of this study is aimed to explore the therapeutic effect of TWEAK on experimental burn wounds, and also the relevant mechanism involving resident cells.

## Materials and Methods

### Murine Model

As described previously (Shen et al., 2013), Fn14-deficient BALB/c mice were generated by using the clustered regularly interspaced short palindromic repeats (CRISPR)/CRISPR-associated (Cas) 9 method (Protocol No. N1-861). Briefly, two guide RNA sequences were designed to interfere with the exon 1 of Fn14 gene. Cas9 mRNA and small-guide RNA were synthesized by using the mMESSAGE mMACHINE T7 Ultra Kit and the MEGAshortscript T7 Transcription Kit, and were then purified with the MEGAclearTM Clean-Up Kit (Thermo Fisher Scientific, Waltham, MA, United States). Both products were injected into the one-cell stage embryos of BALB/c mice. The genotypes were identified by polymerase chain reaction (PCR) in F0 generation mice, which were then hybridized with other mice until Fn14-/- homozygote mice were selected. The mice at age of 10 weeks old were used in the experiments.

Burn wounds were created in mice as previously described (Dai et al., 2011). The mice were anesthetized intraperitoneally with ketamine-xylazine mixture and were shaved on the dorsal surfaces. Two brass blocks, with cross sectional areas of 1 cm × 1 cm, were preheated in boiled water and were then used for scalding hairless skin for 7 s. The success of full-thickness burns was determined by histological examination in preliminary experiments. Each mouse was injected intraperitoneally with 0.5 ml sterile saline to prevent dehydration.

These mice were randomly divided into three groups, with 5 mice in each group. The blank group received no further treatment. The NaCl and TWEAK groups received daily topical administration of normal saline or recombinant murine TWEAK (20 μg/ml, prepared in normal saline; R&D Systems, Minneapolis, MN, United States), respectively.

The mice were then sacrificed on days 0, 3, 7, 14, and 21, followed by the harvest of skin tissues, which were identical to the original burned areas. The harvested skin tissue was equally divided into four parts for further experiments. The Hospital Research Ethics Committee approved all mouse protocols in this study (No. 2016028).

### Immunohistochemistry

Some tissue was routinely processed for paraffin sections. After deparaffinization and rehydration, immunohistochemistry was performed as described previously (Zou et al., 2015). The blocking solution was Dual Endogenous Enzyme Block (DAKO, Glostrup, Denmark). Rabbit anti-TWEAK, Fn14, Iba-1 or CD3 IgG (Abcam, Cambridge, MA, United States) and rabbit anti-epidermal growth factor receptor (EGFR, phosphorylated) IgG (Cell Signaling, Danvers, MA, United States) were used as primary antibodies (2 μg/ml). Polymer-horseradish peroxidase-labeled goat anti-rabbit IgG (DAKO) was secondary antibody (2 μg/ml). Brown-yellow color was developed by using 3, 3′-diaminobenzine-chromogen substrate (DAKO).

Some sections were stained with hematoxylin-eosin or Masson’s trichrome solution. The epidermal thickness was measured with H&E-stained sections. The number of appendage-like structures per mm^2^ area was also counted on these sections. The wound area and histological evaluation were detailed in Supplementary Table S1.

### Cell Culturing

Primary human dermal fibroblasts were purchased from Life Technologies Co. (Carlsbad, CA, United States) and were cultured in medium 106 supplemented with low serum growth supplement (Life Technologies). Some cells were transfected with control (#AM4611) or Fn14 (#135142) siRNA (Life Technologies) (Xia et al., 2015). The transfection efficiency was verified by quantitative real-time PCR (qRT-PCR), which showed a >70% reduction in Fn14 mRNA expression (data not shown).

Prior to TWEAK stimulation, the cells were starved in 2% fetal bovine serum-supplemented medium for 24 h. The cells were stimulated with human recombinant TWEAK (0–250 ng/ml, 0–48 h; Cell Sciences, Canton, MA, United States). Some cells were pretreated with the specific inhibitors of the NF-κB (JSH-23, 20 μM), Wnt/β-catenin (XAV939, 20 nM), EGFR (erlotinib, 10 nM), p38 mitogen-activated protein kinase (MAPK) (TAK-715, 20 nM) and Smad3 (SIS3, 10 μM) signaling pathways (MedChemExpress, Monmouth Junction, NJ, United States) at 24 h before TWEAK stimulation.

### Immunofluorescence

The cells growing on a glass-bottomed culture dish (MatTek, Ashland, MA, United States) were fixed with cold acetone. Cells were then incubated with Alexa Fluor 488-conjugated rabbit IgG targeting alpha-smooth muscle actin (α-SMA) (2 μg/ml; Abcam). Rabbit anti-palladin IgG and Alexa Fluor 647-conjugated goat anti-rabbit IgG were used to detect palladin expression (2 μg/ml; Abcam). After 4′,6-diamidino-2-phenylindole incubation, the cells were observed under a digital confocal microscope (Leica, Wetzlar, Germany).

### Flow Cytometry

Alexa Fluor 488-conjugated rabbit IgG targeting α-SMA (2 μg/ml) was also used for flow cytometry. Similarly, rabbit anti-palladin IgG and Alexa Fluor 647-conjugated goat anti-rabbit IgG were used to detect palladin expression (2 μg/ml). Flow cytometry was performed by using an LSRII instrument (BD Biosciences, San Jose, CA, United States). Data were analyzed using a FlowJo7.6.1 software (Tree Star, Ashland, OR, United States).

### qRT-PCR

Total RNA was extracted from fresh tissues or cell cultures by a PureLink RNA kit (Invitrogen, Grand Island, NY, United States). cDNA was prepared by using a commercial cDNA kit (Applied Biosystems, Carlsbad, CA, United States). qRT-PCR was carried out as described previously (Liu et al., 2016). The primers (Jieqing Biotech Co., Wuhan, China) were detailed in Supplementary Table S2.

### Western Blotting

Protein lysates were routinely extracted from fresh tissues or cell cultures. Western blotting was performed as described previously (Liu et al., 2016). Rabbit IgG to β-actin, regulated on activation normal T cell expressed and secreted (RANTES), monocyte chemoattractant protein (MCP)-1, interferon gamma-induced protein (IP)-10, transforming growth factor (TGF)-β1, EGFR (phosphorylated), MMP-9, hyaluronan synthase 1 (HAS-1), laminin α1, α-SMA or palladin (Abcam) was primary antibody (2 μg/ml). Horseradish peroxidase-conjugated goat anti-rabbit IgG (Abcam) was secondary antibody (2 μg/ml). An ECL chemiluminescence kit (EMD Millipore, Billerica, MA, United States) was used for signal development. The band intensities were measured by ImageJ1.61u software (National Institutes of Health, Bethesda, MD, United States) and were normalized to the values of β-actin bands accordingly.

### Statistical Analysis

All data are expressed as the means ± standard error of the mean (SEM). Statistical analysis was performed using GraphPad Prism version 5.0 (GraphPad Software, La Jolla, CA, United States). Analysis of variance was used for the comparison of more than two groups of variables. On the other hand, two-tailed Student’s *t*-test was used for the comparison of two groups only. Differences were considered significant at *p* < 0.05.

## Results

### Topical TWEAK Promotes Wound Healing in A Murine Burn Model

**FIGURE 1:**
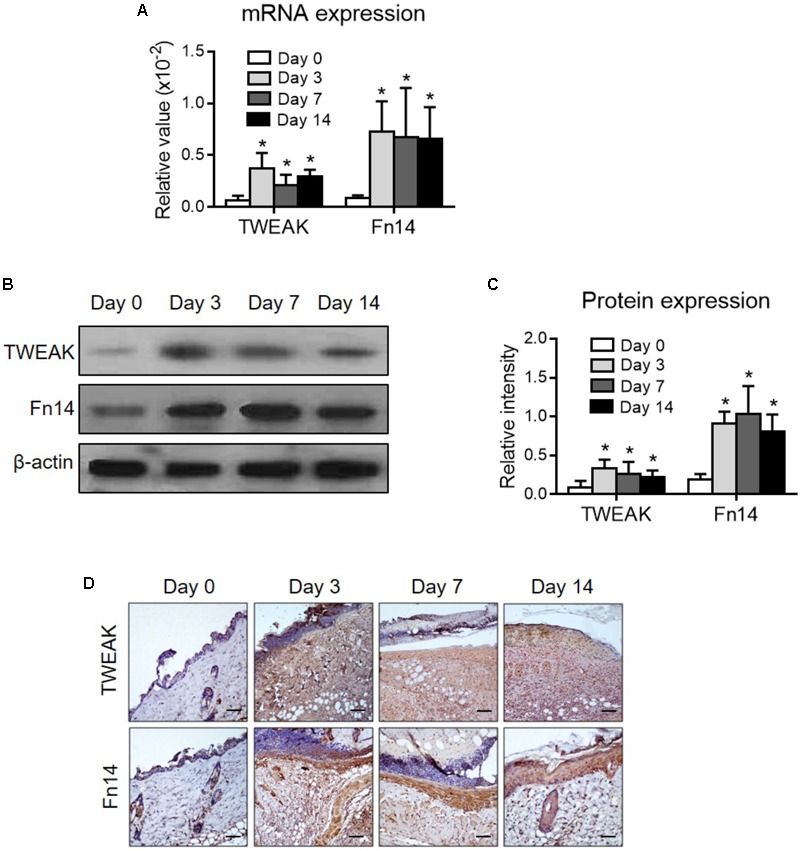
The expression levels of TWEAK and Fn14 in burn wounds. Burn wounds were induced in the wild-type BALB/c mice. **(A)** By quantitative real-time polymerase chain reaction, the mRNA expression levels of TWEAK and Fn14 were determined in lesional tissue at different time points. **(B,C)** By Western blotting, the TWEAK and Fn14 proteins were determined in lesional tissue accordingly. The band intensities were quantitated by ImageJ software. **(D)** By immunohistochemistry, the proteins of TWEAK and Fn14 were detected in skin tissues. Number of mice = 5. In **(A,C)**, ^∗^*p* < 0.05, compared with the day 0 group. There were no significant differences between the other three groups (*p* > 0.05).

**FIGURE 2:**
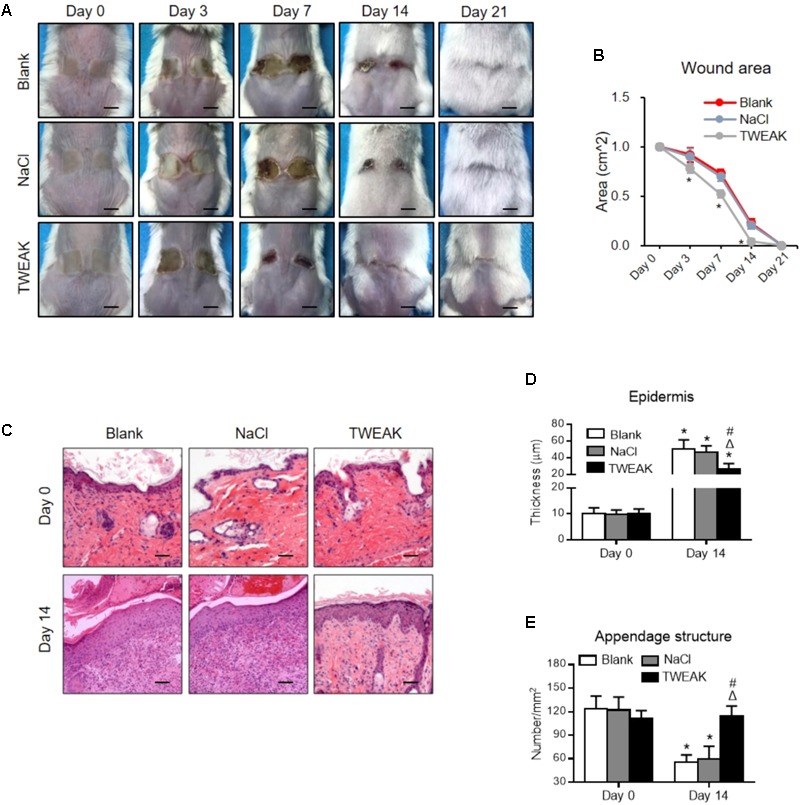
Topical TWEAK promotes the healing of burn wounds. Burn wounds created in the wild-type BALB/c mice were treated with either normal saline (NaCl) or TWEAK solution (20 μg/ml). **(A)** Burn wounds were monitored on days 0, 3, 7, 14, and 21. **(B)** The wound areas were measured in the three groups. ^∗^*p* < 0.05, compared with the blank and normal saline groups. **(C)** Illustrations of hematoxylin-eosin-stained sections. **(D)** The epidermal thickness was determined on these sections, and **(E)** the number of appendage-like structures was counted accordingly. In **(D,E)**, ^∗^*p* < 0.05, compared with day 0 in the same group; Δ*p* < 0.05, compared with blank group on the same day; #*p* < 0.05, compared with normal saline group on the same day. Number of mice = 5. Representative images are shown. Bar = 25 μm.

Firstly, the expression levels of TWEAK and Fn14 were determined in lesional regions of the wild-type mice. By both qRT-PCR and Western blotting, it showed that their expression levels increased significantly after wound creation (*p* < 0.05) (**Figures 1A–C**). By immunohistochemistry, the expressions of TWEAK and Fn14 were also stronger in skin since day 3 (**Figures 1D**). We further explored the effect of exogenous TWEAK on wound healing in this murine model. The skin lesion in wild-type mice was treated topically with recombinant TWEAK (20 μg/ml, 0.5 ml daily). Surprisingly, the wounds in the TWEAK-treated group healed at a faster rate than that in the blank or normal saline controls (**Figures 2A,B**). The TWEAK-treated group had less wound areas than the controls on days 3, 7, and 14 (*p* < 0.05). No significant difference was found between the two controls at any time point (*p* > 0.05). All burn wounds healed completely on day 21, with hairs growing unevenly in original area (**Figures 2A,B**). Furthermore, on day 14, the TWEAK-treated mice exhibited less epidermal thickness but had more appendage-like structures than the controls (*p* < 0.05) (**Figures 2C–E**).

**FIGURE 3:**
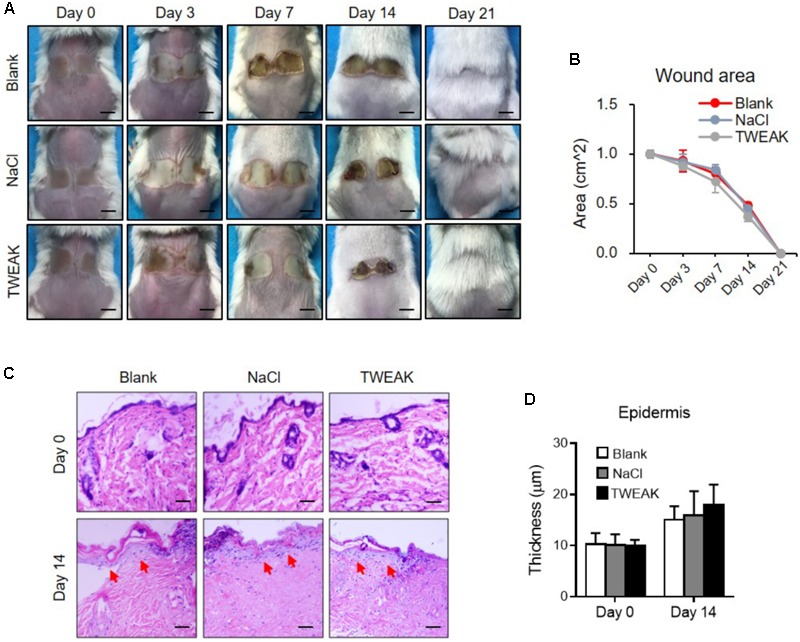
The effect of exogenous TWEAK on burn wounds in Fn14-knochout mice. Burn wounds were induced in mice, followed by the treatments of normal saline (NaCl) or TWEAK solution (20 μg/ml). **(A)** Burn wounds were monitored on day 0, 3, 7, 14, and 21, respectively. **(B)** The areas of skin wounds were calculated in the three groups. **(C)** The images of hematoxylin-eosin-stained sections were shown. Arrows indicated renascent epidermis. **(D)** The epidermal thickness was measured. There were no significant differences in wound area or epidermal thickness between the three groups at the same time point (*p* > 0.05). Number of mice = 5. Representative images are shown. Bar = 25 μm.

The therapeutic experiments were also performed in the Fn14-deficient mice. However, no significant differences in wound area (days 3–21) or epidermal regeneration (day 14) were found among the three experimental groups (*p* > 0.05) (**Figure 3**). All wounds healed on day 21 (**Figure 3**).

### TWEAK Enhances Growth Factor Production and Strengthens Inflammation in Burn Wounds

**FIGURE 4:**
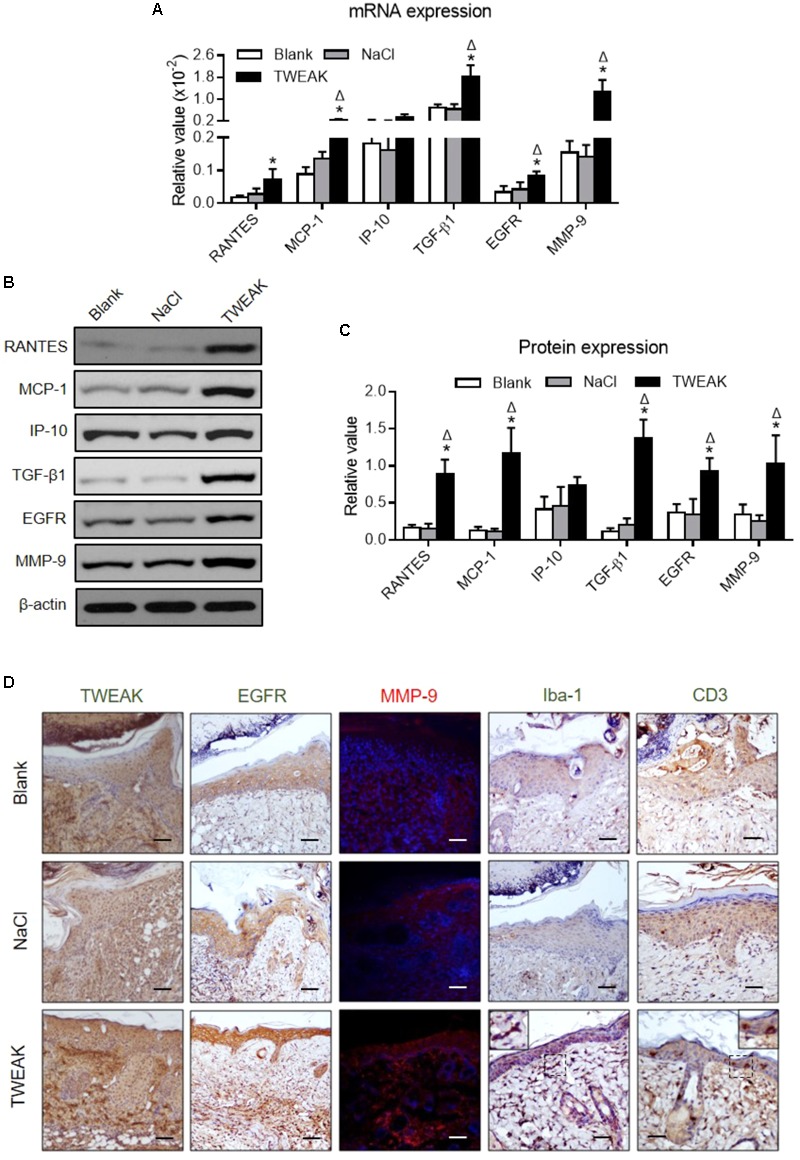
Topical TWEAK enhances protective inflammatory responses in burn wounds. Burn wounds were created in the wild-type BALB/c mice and were treated by either normal saline (NaCl) or TWEAK solution (20 μg/ml). Skin tissue was harvested on day 14. **(A)** The mRNA expression levels of RANTES, MCP-1, IP-10, TGF-β1, EGFR, and MMP-9 were measured in tissue. **(B,C)** The proteins of these molecules were determined by Western blotting. **(D)** The TWEAK, EGFR, and MMP-9 (red) expression, as well as inflammatory cells, were detected by immunohistochemistry or immunofluorescence. In immunofluorescence, the nuclei were stained by 4′,6-diamidino-2-phenylindole (blue). Number of mice = 5. Representative images are shown. Bar = 25 μm. ^∗^*p* < 0.05, compared with blank group; Δ*p* < 0.05, compared with normal saline group.

We also found that, on day 14, the TWEAK-treated mice had higher mRNA expression levels of MCP-1, TGF-β1, EGFR and MMP-9 in wound areas than that in the two controls (*p* < 0.05) (**Figure 4A**). The TWEAK-treated mice had higher RANTES mRNA levels than the blank mice (*p* < 0.05), which were comparable between the TWEAK- and normal saline-treated mice (*p* > 0.05) (**Figure 4A**). However, there were no significant differences in the mRNA level of IP-10 between the three groups (*p* > 0.05) (**Figure 4A**). The proteins of these molecules were further studied through Western blotting at this time point, and it was shown that the TWEAK-treated mice expressed more RANTES, MCP-1, TGF-β1, EGFR and MMP-9 proteins than the two control groups (*p* < 0.05) (**Figures 4B,C**). By immunohistochemistry or immunofluorescence, the TWEAK-treated mice had stronger TWEAK, EGFR, and MMP-9 staining, accompanied by more Iba-1 or CD3 positive cells in wound areas (**Figure 4D**).

### TWEAK Amplifies Extracellular Matrix Synthesis in Burn Wounds

**FIGURE 5:**
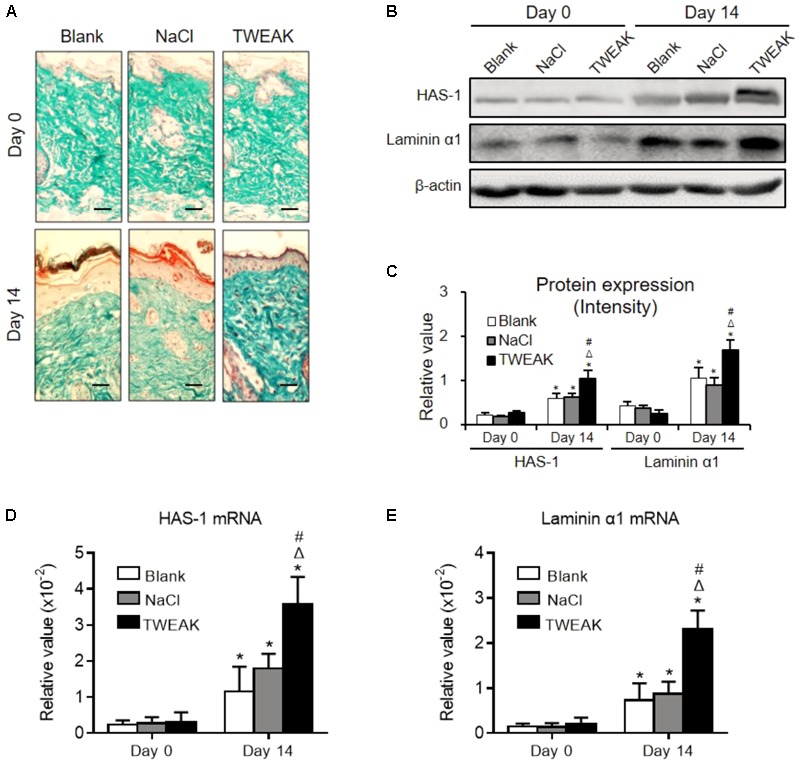
Topical TWEAK promotes extracellular matrix synthesis in burn wounds. Burn wounds created in the wild-type BALB/c mice were treated with either normal saline (NaCl) or TWEAK solution (20 μg/ml). **(A)** The distribution of cutaneous collagen was detected by Masson’s trichrome staining. **(B,C)** The HAS-1 and laminin α1 proteins were determined by Western blotting. The mRNA expression levels of HAS-1 **(D)** and laminin α1 **(E)** were determined accordingly. ^∗^*p* < 0.05, compared with day 0 in the same group; Δ*p* < 0.05, compared with blank group on the same day; #*p* < 0.05, compared with normal saline group on the same day. Number of mice = 5. Representative images are shown. Bar = 25 μm.

Cutaneous collagen was evaluated in Masson’s trichrome-stained sections, in which a similar collagen fraction distribution was demonstrated between the two control groups (day 14). However, the TWEAK-treated mice had stronger collagen staining than that of the controls on day 14 (**Figure 5A**). Moreover, the HAS-1 and laminin α1 protein expression levels, as well as their mRNA expression levels, were higher in the TWEAK-treated mice on day 14 (*p* < 0.05) (**Figures 5B–E**).

### TWEAK/Fn14 Signaling Favors Myofibroblastic Differentiation of Dermal Fibroblasts

**FIGURE 6:**
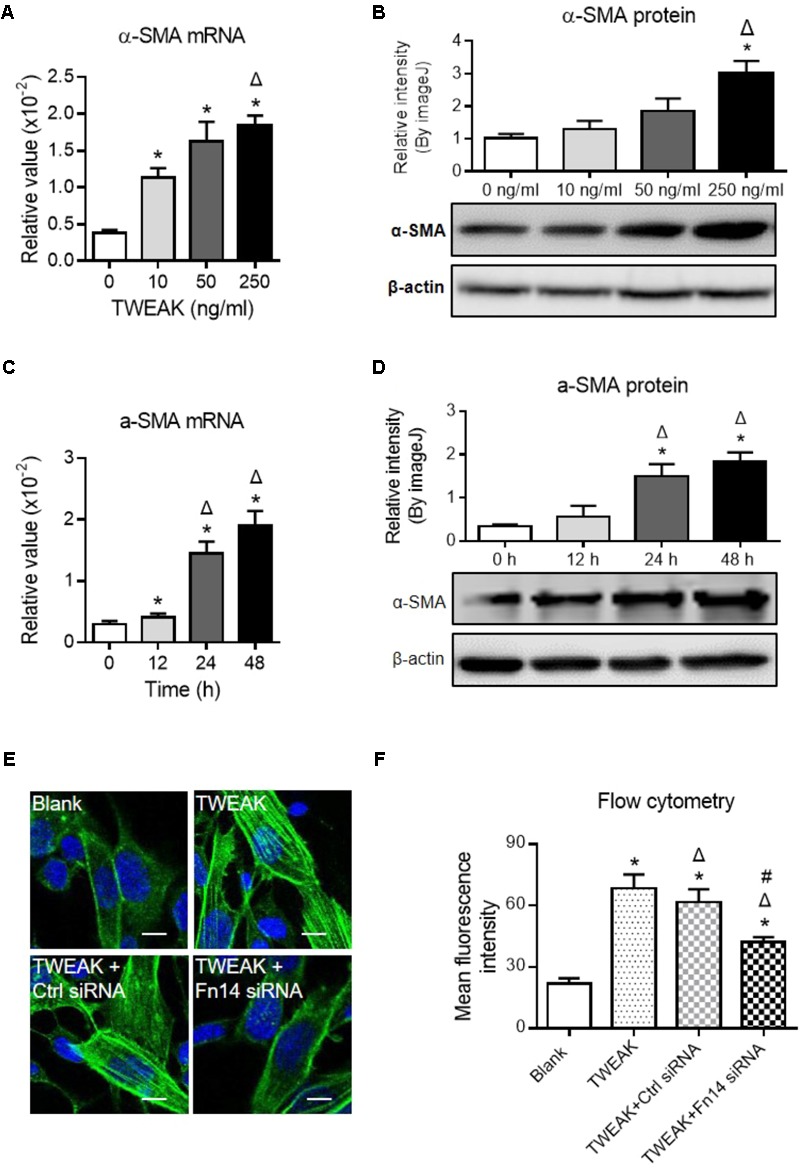
TWEAK upregulates α-SMA expression in dermal fibroblasts. Human dermal fibroblasts *in vitro* received TWEAK stimulation. **(A,B)** The mRNA and protein expression levels of α-SMA were measured after 48-h stimulation. ^∗^*p* < 0.05, compared with the 0 ng/ml group; Δ*p* < 0.05, compared with the 10 ng/ml group. **(C,D)** The mRNA and protein expression levels of α-SMA were measured after 250 ng/ml stimulation (0–48 h). ^∗^*p* < 0.05, compared with the 0 h group; Δ*p* < 0.05, compared with the 12 h group. **(E,F)** By immunofluorescence and flow cytometry, the expression of α-SMA was detected in cells that received siRNA transfection plus TWEAK stimulation (250 ng/ml, 48 h). ^∗^*p* < 0.05, compared with blank group; Δ*p* < 0.05, compared with TWEAK alone group; #*p* < 0.05, compared with TWEAK + control siRNA group. *n* = 3. Representative images are shown. Bar = 5 μm.

Myofibroblastic differentiation of dermal fibroblasts plays a central role in wound healing (Velasquez et al., 2013). The effect of TWEAK/Fn14 interaction on such differentiation process was studied by determining the expression of α-SMA, which is a key differentiation marker. It showed that both mRNA and protein expression levels of α-SMA in dermal fibroblasts were increased with TWEAK stimulation in a dose-dependent manner (0–250 ng/ml) (*p* < 0.05) (**Figures 6A,B**). Moreover, the mRNA and protein expression levels of α-SMA exhibited time-dependent (0–48 h) variation tendencies upon TWEAK stimulation (*p* < 0.05) (**Figures 6C,D**). Dermal fibroblasts were pre-transfected with Fn14 siRNA and were then stimulated with TWEAK (250 ng/ml, 48 h). Through immunofluorescence and flow cytometry, transfection with Fn14 siRNA, but not with control siRNA, partially abrogated TWEAK-induced upregulation of α-SMA protein (*p* < 0.05) (**Figures 6E,F**).

**FIGURE 7:**
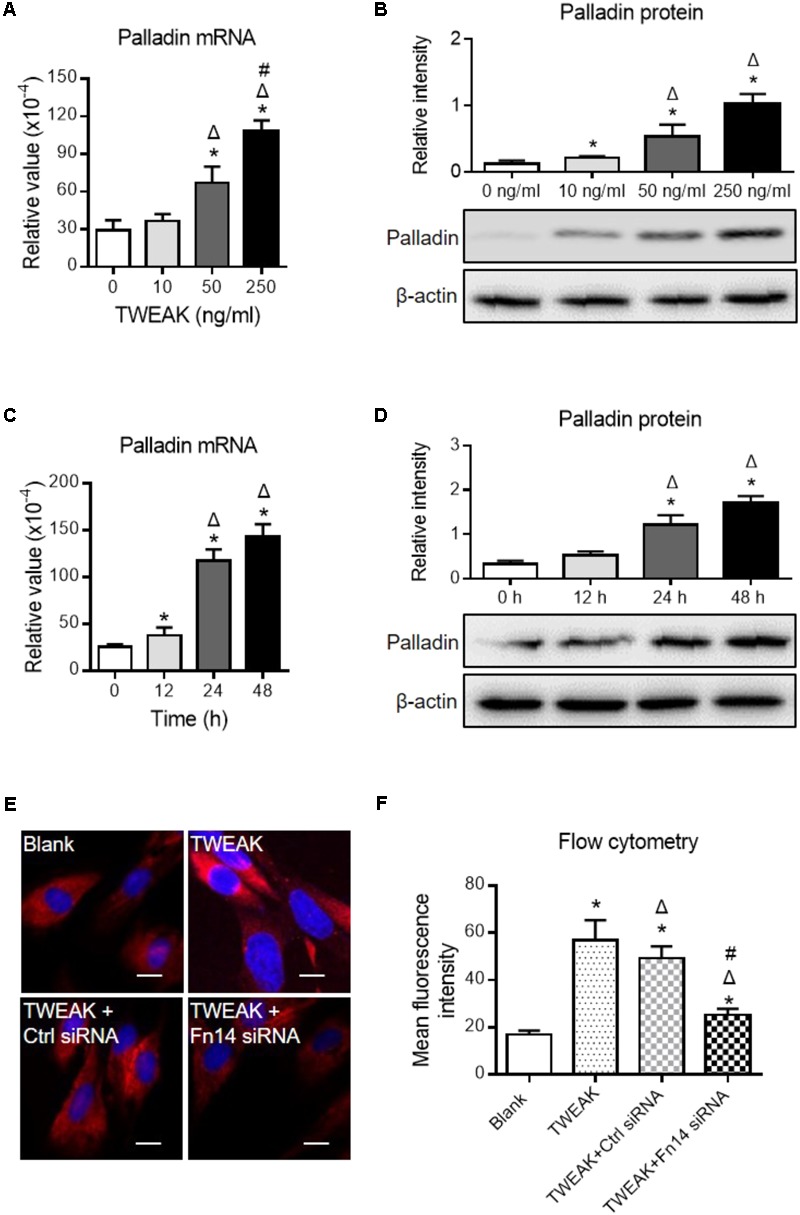
TWEAK enhances the expression of palladin in human dermal fibroblasts. Cells were cultured *in vitro*, followed by TWEAK stimulation. **(A,B)** The mRNA and protein expression levels of palladin were measured after 48-h stimulation. ^∗^*p* < 0.05, compared with the 0 ng/ml group; Δ*p* < 0.05, compared with the 10 ng/ml group; #*p* < 0.05, compared with the 50 ng/ml group. **(C,D)** The mRNA and protein expression levels of palladin were measured after 250 ng/ml TWEAK stimulation (0–48 h). ^∗^*p* < 0.05, compared with the 0 h group; Δ*p* < 0.05, compared with the 12 h group. **(E,F)** By immunofluorescence and flow cytometry, the palladin expression was detected in cells that received siRNA transfection plus TWEAK stimulation (250 ng/ml, 48 h). ^∗^*p* < 0.05, compared with blank group; Δ*p* < 0.05, compared with TWEAK alone group; #*p* < 0.05, compared with TWEAK + control siRNA group. *n* = 3. Representative images are shown. Bar = 5 μm.

We also determined palladin in dermal fibroblasts, which is another important marker for myofibroblast differentiation (Ronty et al., 2006), and we found that both mRNA and protein expression levels of palladin increased with the addition of TWEAK (*p* < 0.05) (**Figures 7A–D**). By immunofluorescence and flow cytometry, transfection with Fn14 siRNA abrogated the upregulation of palladin induced by TWEAK stimulation (*p* < 0.05) (**Figures 7E,F**).

**FIGURE 8:**
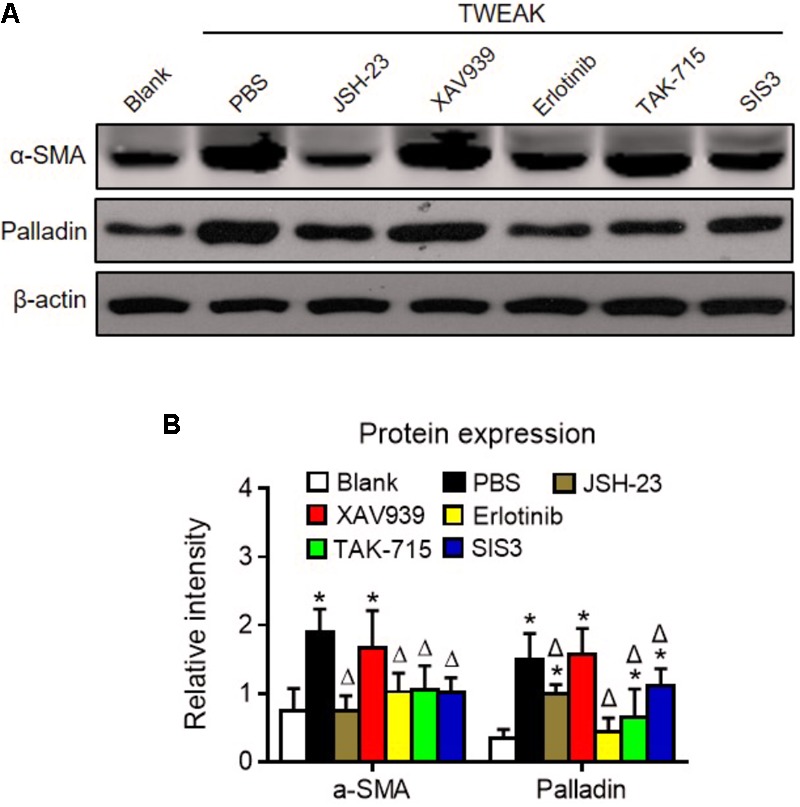
Specific inhibitors abrogate TWEAK upregulation of α-SMA and palladin in dermal fibroblasts. Human dermal fibroblasts were cultured *in vitro* and received TWEAK stimulation (250 ng/ml, 48 h). **(A)** By Western blotting, the α-SMA and palladin proteins were determined in cells that were pretreated with NF-κB (JSH-23), Wnt/β-catenin (XAV939), EGFR (erlotinib), p38 MAPK (TAK-715), and Smad3 (SIS3) inhibitors and were followed by TWEAK stimulation. **(B)** The band intensities were quantitated by ImageJ software. The ^∗^*p* < 0.05, compared with blank group; Δ*p* < 0.05, compared with the PBS group. *n* = 3. Representative images are shown. Bar = 5 μm.

Dermal fibroblasts were further pretreated with specific inhibitors of the nuclear factor (NF)-κB (JSH-23), Wnt/β-catenin (XAV939), EGFR tyrosine kinase (erlotinib), p38 mitogen-activated protein kinase (p38 MAPK; TAK-715), and Smad3 (SIS3) pathways. Interestingly, these inhibitors, except XAV939, significantly reduced both α-SMA and palladin expression that was enhanced by TWEAK (*p* < 0.05) (**Figure 8**).

## Discussion

In this study, we found that topical administration of TWEAK accelerates the healing of experimental burn wounds. TWEAK strengthens inflammatory responses, enhances growth factor production, and amplifies extracellular matrix synthesis in lesional tissue. Moreover, TWEAK/Fn14 interaction induces myofibroblastic differentiation of dermal fibroblasts *in vitro*. Such effect of TWEAK on dermal fibroblasts involves the NF-κB, EGFR, p38 MAPK and Smad3 pathways. Therefore, TWEAK exhibits therapeutic effect on burn wounds, possibly involving the regulation of dermal fibroblasts.

Since TWEAK is mainly produced by infiltrating cells such as macrophages and monocytes (Liu et al., 2017a), its expression level is very low in normal tissue. Fn14 is also slightly expressed in normal cells. However, their expression levels are upregulated remarkably in inflamed tissue. TWEAK/Fn14 activation may even induce loss of junctional proteins, filtration barrier distribution, and irreversible tissue deconstruction (Berzal et al., 2015; Wen et al., 2015; Xia et al., 2015; Wilhelm et al., 2016). Recently, it was found that subcutaneous injection of recombinant TWEAK induces skin inflammation as well as epidermal and dermal hyperplasia (Sidler et al., 2017). In this study, we confirmed that topical administration of TWEAK (20 μg/ml, daily) does not exacerbate skin damage but accelerates the repair of burn wounds. Hence, the interplay between TWEAK and Fn14 may also be favorable during tissue injuries – depending on the strength of TWEAK/Fn14 activation and specific tissue responses.

The TWEAK-regulated downstream cytokines or receptors include RANTES, MCP-1, IP-10, and EGFR (Xia et al., 2012; Rayego-Mateos et al., 2013; Wen et al., 2013; Liu et al., 2017c), which play a synergetic role in collagen synthesis and tissue remodeling (Kim et al., 2014; Nosbaum et al., 2016). TGF-β1 also activates Fn14 expression through the transcription factor Smad4, and Fn14 activation further increases extracellular matrix synthesis in dermal fibroblasts (Chen et al., 2015). So, the upregulation of these cytokines directly verifies the activation of TWEAK/Fn14 signals and indirectly reflects the production of extracellular matrix. In the present study, topical TWEAK increases the expression levels of these cytokines and EGFR in burn wounds. Moreover, another downstream molecule MMP-9 is robustly expressed in TWEAK-treated wounds. MMP-9 plays an important role during repair processes of skin injuries, participating in cell migration and remodeling events (Kopcewicz et al., 2017). Furthermore, more macrophages (Iba-1 positive) and T lymphocytes (CD3 positive) infiltrate in lesional tissue of this murine model upon TWEAK administration. Taken together, these findings demonstrated that exogenous TWEAK enhances regional synthesis of cytokines or molecules, as well as recruitment of immune cells, that function vitally in skin wound repair.

Extracellular matrix plays a pivotal role in fibroblast activation, wound contraction, and tissue remodeling during skin wound repair (Qing, 2017). Collagen is the main component of extracellular matrix. We found that collagen is more expressed in burn wounds after topical TWEAK administration. Both HAS-1 and laminin α1 are important constituents of the extracellular matrix. HAS-1 is actively produced during tissue repair and provide an environment for cell migration and blood vessel growth. Laminin exhibits protective function in the healing of burn wounds through favoring the proliferation, migration, and adherence of skin cells (Olczyk et al., 2012). we observed that topical TWEAK upregulates the expression of HAS-1 and laminin α1 in lesional tissue of experimental burn wounds. Therefore, TWEAK/Fn14 activation definitely enhances extracellular matrix synthesis in burn wounds.

Dermal fibroblasts are critical in the healing of burn wounds. Actually, TWEAK regulates basic function of dermal fibroblasts, including the secretion of interleukin-19 and thymic stromal lymphopoietin (Sidler et al., 2017). Interleukin-19 can promote skin wound healing by increasing the expression of the fibroblast or keratinocyte growth factor (Sun et al., 2013). Thymic stromal lymphopoietin is a Th2-promoting cytokine contributing to the progression of skin fibrosis (Oh et al., 2011). In this study, we further provided strong evidences that TWEAK/Fn14 interaction induces myofibroblast-like differentiation of dermal fibroblasts, reflected by upregulation of α-SMA and palladin. Hence, such effect of TWEAK on dermal fibroblasts partially explains the mechanism underlying its therapeutic role in burn wound healing. In addition, such regulatory effect of TWEAK on dermal fibroblasts is mediated by the NF-κB, EGFR, p38 MAPK, and Smad3 pathways. Other investigators demonstrated that TWEAK promotes the proliferation and myofibroblast differentiation of renal fibroblasts in a NF-κB- and Ras-dependent manner (Ucero et al., 2013; Gomez et al., 2016). Therefore, the precise chains of signaling transduction deserves further investigation in next studies.

## Conclusion

Topical application of TWEAK strengthens inflammatory responses, cytokine production, and extracellular matrix synthesis, which synergistically accelerate the healing of experimental burn wounds. TWEAK can promote the myofibroblast differentiation of dermal fibroblasts. Future studies should be focused on the relevant mechanism by which TWEAK regulates other skin cells, and on the approaches how to improve therapeutic effect of TWEAK treatment.

## Author Contributions

JL participated in the design of the study, and performed the experimental work. LP performed the animal experiments. YL, KW, SW, XW, and QL carried out some experiments. YX and WZ conceived and designed the study and prepared the manuscript. All the authors read and approved the final manuscript.

## Conflict of Interest Statement

The authors declare that the research was conducted in the absence of any commercial or financial relationships that could be construed as a potential conflict of interest.
