# Supplementary figures and images for: Topical TWEAK Accelerates Healing of Experimental Burn Wounds in Mice
Source: Front Pharmacol. 2018 Jun 21;9:660. doi: 10.3389/fphar.2018.00660 (PMC6021523; doi:10.3389/fphar.2018.00660)

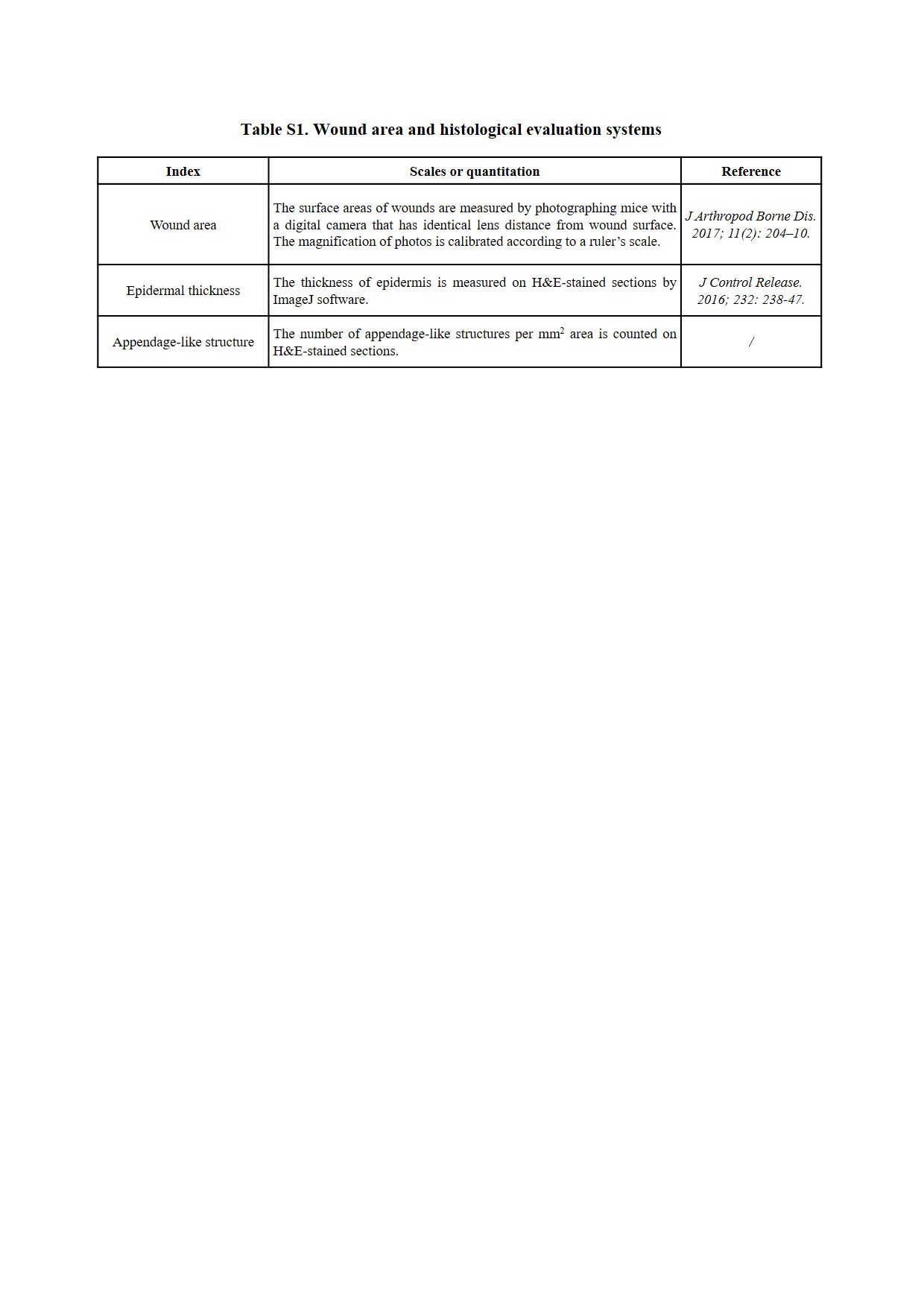

Supplement: Supplementary file 1 [file Image_1.JPEG]

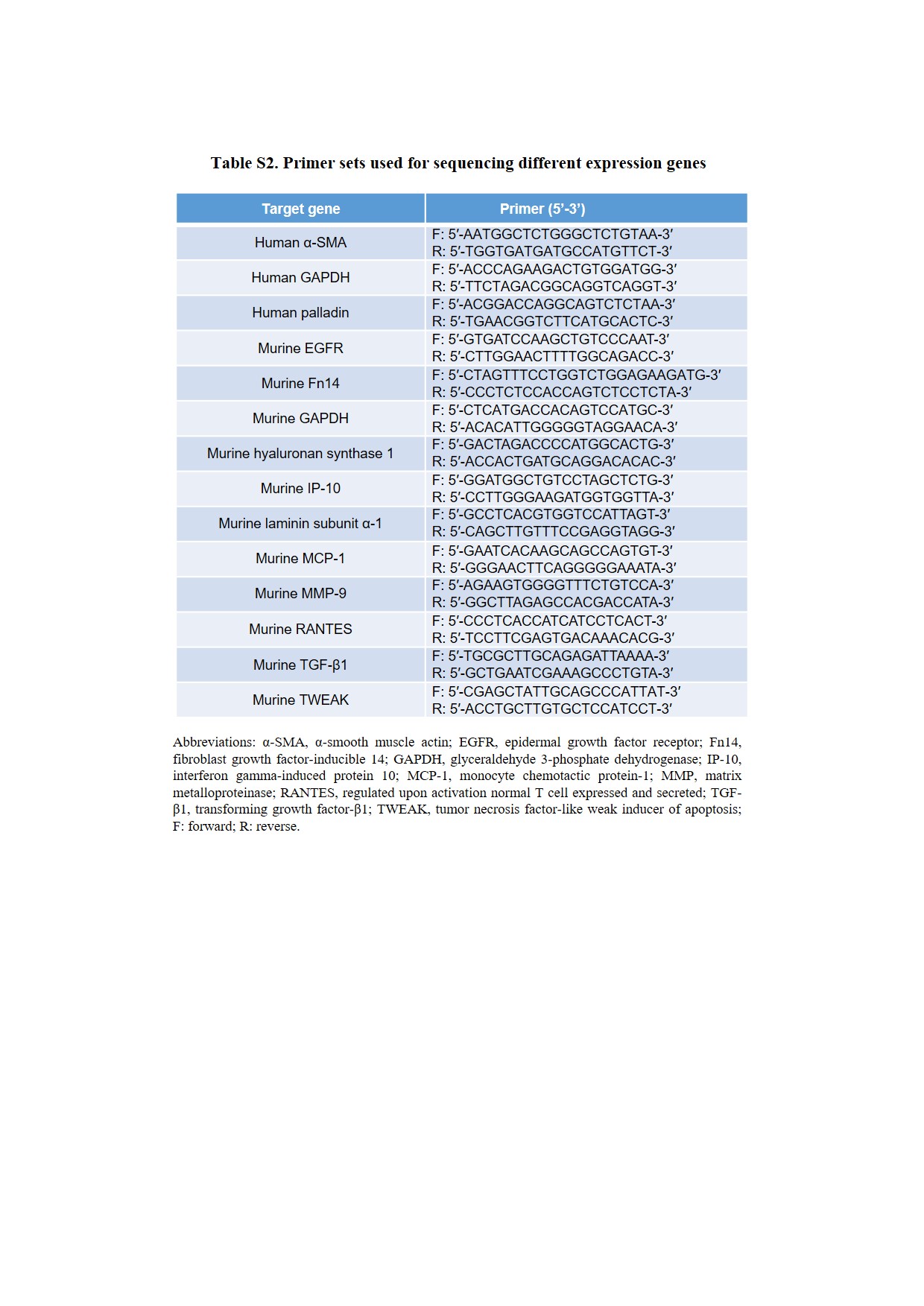

Supplement: Supplementary file 2 [file Image_2.JPEG]
